# A standardized model to study effects of varying 24-h colostrum dose on postnatal growth and development

**DOI:** 10.1093/tas/txaa212

**Published:** 2020-11-19

**Authors:** Aridany Suárez-Trujillo, L Kirsten Senn, Kelsey Teeple, Theresa M Casey, Kara R Stewart

**Affiliations:** Department of Animal Sciences, Purdue University, West Lafayette, IN

**Keywords:** colostrum, intake, model, piglet

## Abstract

Survival, feed efficiency, growth, and fertility of swine are dependent on colostrum intake in the first 24 h after birth. This study determined the effects of three doses of a homogeneous colostrum sample on 24-h body weight, rectal temperature (RT), immunocrit, and growth and survival to postnatal day (PND) 7. Three female piglets were selected from eight litters (*n* = 24 piglets) at birth, removed from their litter, and bottle-fed 10% (COL10, *n* = 8), 15% (COL15, *n* = 8), or 20% (COL20, *n* = 8) colostrum based on birth weight over 12 bottle feedings every 2 h. At 24 h, piglets were weighed, RT recorded, and blood was collected to measure immunocrit. Piglets were returned to the litter of origin, and weight was measured daily until PND 7. Colostrum dose had an overall effect on weight gain at 24 h, RT, immunocrit, and growth to PND 7 (*P* < 0.05). Piglets in the 20% BrW colostrum group had greater weight gain, RT, and immunocrit at 24 h than COL10 piglets (*P* < 0.05), but these variables were not different between COL15 and the other treatments. Despite no difference in average daily gain after being returned to their litters, the greater weight (*P* < 0.05) in COL20 compared to COL10 and COL15 was sustained over 7 d. Seven piglets in each treatment survived to PND 7. This model using standardized doses of a homogeneous colostrum sample enables controlled studies aimed at understanding the role of 24-h colostrum intake on piglet development.

## INTRODUCTION

Colostrum is essential for piglet survival and development. Colostrum is the first secretion of the mammary gland and has a higher concentration of fat, proteins, and immunoglobulins than mature milk (Theil et al., 2014). Sufficient colostrum consumption is needed to provide piglets with enough energy to survive and perform thermogenesis until copious milk production begins. Colostrum intake is also essential for the passive transfer of maternal immunoglobulins to neonatal pigs (Theil et al., 2014). Inadequate colostrum consumption results in low weight gain, hypothermia, increased probability of crushing by the sow, and increased death in the first few days after birth. Bioactive components of colostrum induce the development of somatic tissues, and the amount consumed of such bioactive components (i.e., immunoglobulins or relaxin) has long-term impacts on feed efficiency, carcass composition, and reproduction (Bartol and Bagnell, 2012; Bartol et al., 2013).

Studying the impacts of varying colostrum intakes in piglets poses several challenges. Studies aimed at understanding the influence of maternal traits on piglet growth (Jin et al., 2017; Laws et al., 2018; Jiarpinitnun et al., 2019) usually allow piglets to remain with the sow and nurse ad libitum and estimate colostrum intake using the weigh-suckle-weigh procedures or changes in body weight during the first 24 h (Devillers et al., 2004). In this herd-level type of analysis, piglets are allowed to nurse in their natural environment and there is no control of the variation in colostrum production and composition from the sow; hence, it lacks in quantitative relationships (Foisnet et al., 2010; Kielland et al., 2015). Studies aimed at determining the effects of feeding bioactive components on piglet development have used a control group that received no colostrum (Fiorotto et al., 2000; Ashley et al., 2018). Negative control groups were fed a milk replacer or other commercially available colostrum supplement, such as bovine colostrum (De Vos et al., 2014). However, there is a high mortality rate in piglets that receive no colostrum, despite milk replacement, resulting in most of these studies being relatively short in duration [e.g., 24–48-h studies (Fiorotto et al., 2000; Ogawa et al., 2016; Harlow et al., 2019)]. Moreover, milk replacers and supplements, even if formulated to match the gross composition of fat, lactose, and protein, cannot completely match amino acids, lipids, and other bioactive molecules of pig colostrums and, thus, potentially induce distinct cellular mechanisms.

Therefore, there is a need to develop a research model to study the impact of colostrum intake on piglet growth and development that reduces contributors to variation from the sow while minimizing piglet mortality in the first few days of postnatal life. It has been established that 24-h colostrum intake is positively related to postnatal day (PND) 1 immunocrit, body temperature, and long-term growth trajectory (Devillers et al., 2011). A neonatal intake of less than 290 g of colostrum decreased postnatal growth rate. Taking the average birth weight into account, this data indicated that piglets that ingested at least 20% of birth weight in terms of colostrum intake over the first 24 h postnatal were on a higher plane of growth than piglets that ingested less. Using these guidelines, we designed a study to determine the effect of 10%, 15%, and 20% of birth body weight colostrum intake over the first 24 h postnatal on the percentage of immunocrit, rectal body temperature, and growth and survival to PND 7 after returning to the dam.

## MATERIALS AND METHODS

### Animal and Study Design

The protocols were reviewed and approved by Purdue University’s Institutional Animal Care and Use Committee (Protocol #1907001920) prior to the start of the study. Animals used for the study were from the Purdue University Animal Science Research and Education Swine facility, and farm protocols regarding farrowing and postnatal care were followed. Three female piglets (gilts) with body weights between 1.2 and 1.8 kg were selected from eight litters of multiparous sows (3.25 ± 1.67 parities) at birth (T0) over four consecutive days in March 2020, resulting in 24 study animals. The weight range was selected to limit the effect of birth size on postnatal growth. To prevent the initiation of suckling from the sow, piglets were removed from the farrowing crate immediately after birth, towel dried and moved to a temperature controlled nursery that was maintained at 40 °C. Three gilts within eight different litters were selected, ear tagged for identification, weighed, and assigned to one of three treatment groups matched according to birth weight by alternating the heavier and lighter piglet across the treatments. The three treatment groups were: 1) 20% colostrum (COL20; *n* = 8); 2) 15% colostrum (COL15; *n* = 8); and 3) 10% colostrum (COL10; *n* = 8) administered over the first 24 h postnatal, and the average birth body weights were 1.51 ± 0.20 kg in COL20; 1.45 ± 0.27 kg in COL15; and 1.48 ± 0.16 kg in COL10. In the nursery, piglets were grouped housed in pens by litter mates to ensure that piglets were fed at the same time, avoiding disturbing piglets from other litters. The nursery was located in a separate building and pens were 50- × 40-cm sized, with slotted floors and heated to 40 °C.

Colostrum doses were calculated into 12 equal-sized feedings for the piglet to receive via bottles at 2-h intervals. Colostrum feeding began within 2 h of birth using infant bottles and were completed at 24 h postnatal. Colostrum doses were weighed and then warmed in a water bath (40 °C) for 10 min before being presented to the piglets. If the piglet refused the bottle, leftover colostrum was weighed, recorded, saved in the bottle, and refrigerated to be added to the next scheduled dose of colostrum. After the completion of 24-h colostrum bottle feeding, piglets were returned to their birth litters, which were standardized on the second day after birth to 12–14 piglets per sow.

Piglets used for this trial were part of a larger study, for which COL20 and COL10 groups were administered a saline solution of deuterium oxide (0.9% NaCl in D_2_O, 20 mL/kg of BW) via intraperitoneal injection (IP) at birth. The COL15 group was administered saline (0.9% NaCl in H_2_O) IP at birth. At 24 h postnatal, after body temperature and blood samples were collected, and then daily to PND 7, piglets were orally gavaged with 10 mL/kg of BW deuterium oxide (COL2 and COL10) or water (COL15).

### Collection and Preparation of Colostrum

Colostrum was collected from multiple sows (~250) over the course of 7 mo. Roughly 50 mL of colostrum was collected from each sow during active farrowing, frozen, and stored at −80 °C until the day prior to the start of the study, when colostrum was thawed in the fridge overnight. After thawing, colostrum samples were combined and thoroughly mixed by inverting to create a uniform colostrum mixture. The uniform colostrum mix was stored at 4 ºC until piglet feeding.

### Blood Sampling, Immunocrit Analysis, and Rectal Body Temperature

All measurements and sample collection were performed before the dosing of deuterium oxide or water in order to minimize the effect of stress on variables being measured. Blood was collected from gilts at 24 h postnatal via jugular venipuncture using a 22-gauge × 2.5-cm needle and vacutainer tubes, while the piglet was held in dorsal recumbency. Up to ~1.5 mL of blood was collected into a 2-mL potassium-EDTA-coated blood tube (BD367841, BD, Franklin Lakes, NJ). Blood was immediately centrifuged at 2,000 × *g* for 15 min to separate the plasma fraction (E8 Centrifuge, LW Scientific Inc., Lawrenceville, GA). Plasma was divided into 150-µL aliquots in separate 1.5-mL microtubes to measure immunocrit (Vallet et al., 2013). Immunocrit is a method to evaluate the efficiency of immune passive transfer in piglets, allowing for on-farm determination of immune status (Vallet et al., 2013). To measure immunocrit, a mixture of 150 µL plasma and 150 µL of 40% ammonium sulfate solution [(NH_4_)_2_SO_4_, Sigma-Aldrich, St. Louis, MO] was made and inverted several times to mix and prevent precipitation. Three nonheparinized hematocrit tubes (22-362574, Fisherbrand, Waltham, MA) were filled with the plasma:ammonium sulfate solution by inserting one end of the hematocrit tube into the solution and allowing it to fill to 75% capacity using capillary action before sealing the bottom of the tube with clay. Tubes were then centrifuged at 12,000 × *g* for 10 min (Microhematocrit centrifuge LWS-M24, LW Scientific Inc., Lawrenceville, GA). A digital caliper (Tool Shop 6” Stainless Steel Digital Caliper, Menards, Eau Claire, WI) was used to measure the length of precipitate and total length of solution. Immunocrit was expressed as the percentage of precipitate length of total length.

Rectal temperature was taken at 24 h postnatal using a digital thermometer (FlashCheck Jumbo Display Auto-Cal Needle Probe Thermometer, Model 11063, Deltatrak, Pleasanton, CA). Piglets were weighed on a digital scale at birth, 24 h postnatal, and daily at 0800 h to PND 7 and used to calculate average daily gain (ADG).

### Statistical Analysis

Statistical analysis was performed using MIXED procedure in SAS (version 9.4; Cary, NC), including the Tukey method for multiple means comparison. Treatment (COL10, COL15, and COL20) was considered as the main effect for birth weight, 24 h weight gain, rectal temperature, and immunocrit. Treatment, day, and their interaction were considered as main effects for the analysis of the repeated measures of body weight and ADG data collected over the first 7 d postnatal. *P*-values < 0.05 were considered significantly different, whereas *P*-values between >0.05 and <0.1 were considered a tendency.

## RESULTS

As designed, birth weights were similar across treatments (Table 1; COL20 1.51 ± 0.20 kg; COL15 1.45 ± 0.27 kg; COL10 1.48 ± 0.16 kg; *P* = 0.838). Average daily gain in the first 24 h (Table 1; *P* < 0.001), rectal temperature (Table 1; *P* = 0.007), and immunocrit (Table 1; *P* = 0.026) were different among treatments. Post hoc analysis indicated that the individual weight gain of COL10 (8.8 ± 24.9 g) gilts was lower than COL15 (86.3 ± 85.2 g, *P* < 0.001) and COL20 piglets (136.3 ± 54.8 g; *P* < 0.001). Body temperature at 24 h postnatal was greater in COL20 piglets (39.30 ± 0.11 °C) than in COL15 (38.84 ± 0.45 °C; *P* = 0.017) and COL10 (38.81 ± 0.26 °C; *P* = 0.012) piglets. COL20 piglets (0.028 ± 0.010) had greater immunocrit (*P* = 0.021) than COL10 piglets (0.017 ± 0.004), whereas COL15 (0.024 ± 0.006) were not different from either treatment.

**Table 1.** Effect of feeding 20% (COL20), 15% (COL15), and 10% (COL10) of birth body weight of colostrum on weight gain, body temperature, and immunocrit at 24 h postnatal

	COL20	COL15	COL10	SEM^*a*^	*P*-value^*b*^
Birth weight, kg	1.51	1.45	1.48	0.27	0.838
ADG at 24 h, g	136.3a	86.3a	8.8b	85.2	<0.001
Body temperature, °C	39.30a	38.84b	38.81b	0.45	0.007
Immunocrit	0.028a	0.024ab	0.017b	0.010	0.021

^*a*^Largest standard errors among treatments.

^*b*^ *P*-value for the treatment main effect.

a,bValues with different letters were statistically different (*P* < 0.05) after Tukey’s post hoc test.

**Figure 1.**
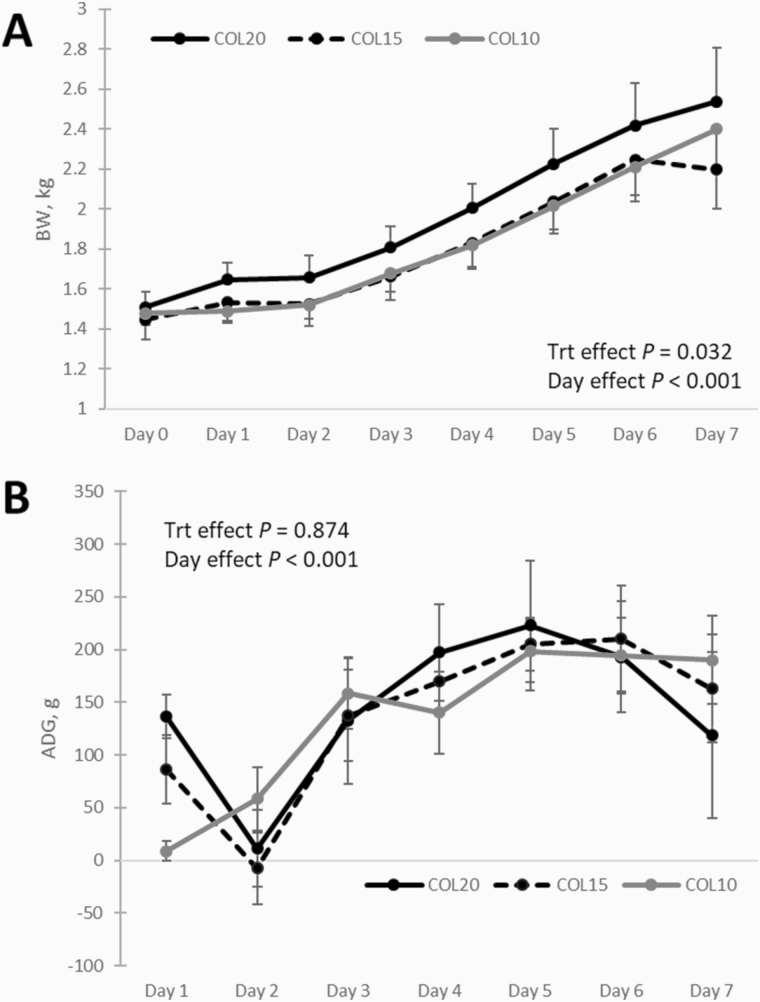
Body weight (A) and ADG (B) development over 7 d postnatal. Piglets were collected at birth from eight litters (three from each litter) and assigned to be fed 20% (COL20, solid black), 15% (COL15, dashed black), or 10% (COL10, solid gray) of their birth weight of colostrum over the first 24 h postnatal. After 24 h, piglets were returned to their litter of origin. Every morning at 0800 h, piglet weights were recorded and ADG was calculated.

After returning to their birth litter at 24 h of age, one piglet in each treatment died by crushing. The COL10 piglet died on D3, COL15 piglet on day 6, and COL20 piglet on day 2. Treatment had an effect on body weight up to PND 7 (*P* = 0.032; Fig. 1A). Post hoc analysis determined that COL20 piglets (1.96 ± 0.53 kg) tended to have greater body weight than COL15 (1.82 ± 0.48 kg, *P* = 0.052) and COL10 (1.81 ± 0.45, *P* = 0.067). Average daily gain (Fig. 1B) over 7 d postnatal did not vary among treatments (*P* = 0.874; COL20 141.8 ± 147.9 g/d; COL15 137.3 ± 115.3 g/d; COL10 133.0 ± 112.3 g/d).

## DISCUSSION

Here, we established that gilts fed for colostrum intakes of 10% or 15% of birth body weight gained significantly less weight than those fed for 20% body weight intake over the first 24 h postnatal. The differences in weight by colostrum dose were sustained across the first 7 d postnatal despite no difference in ADG between PND 2 and 7 among the treatments. By feeding a pooled sample of colostrums, the effect of variation in colostrum quality was controlled and enabled the analysis of colostrum quantity alone on 24 h immunocrit, body temperature, and postnatal growth rate. While this study was not powered to investigate preweaning mortality, the development of an animal model to investigate the long-term impacts of low colostrum intake would require the animals to consume enough colostrum to survive through weaning or beyond. The low dose of colostrum (10% of birth body weight) was sufficient for neonatal survival to weaning after return to the litter of origin, while resulting in a significantly lower piglet body weight through PND 7 compared to the high dose of colostrum (20% birth body weight). The impact of varying birthweight on postnatal survival and growth rate was also minimized by limiting range in birth weight to between 1.2 and 1.8 kg. This is an important finding and supports the use of the model system in studies designed to understand how 24-h postnatal colostrum intake establishes long-term growth rate, feed efficiency, carcass composition, and fertility. Moreover, using different quantities of pooled colostrum samples, as opposed to using milk replacers as null treatment, eliminates the potential that components of replacer formula, like fats (Harlow et al., 2019), could be affecting neonatal development.

Immunocrit in blood plasma positively correlates with the amount of colostrum consumed and reflects the neonate’s ability to absorb large bioactive molecules through passive transfer in the small intestine (Ogawa et al., 2016). COL20 had the highest immunocrit, indicative of a higher level of immunoglobulins in their blood at 24 h postnatal compared to COL10. This is consistent with herd-level data that found that lower intake of colostrum was related to lower 24-h immunocrit (Devillers et al., 2011). At birth, piglets have little glycogen stores and no subcutaneous fat; therefore, they rely on energy from colostrum consumption to provide energy for shivering to increase body temperature, as well as to perform maintenance functions (such as suckling and digestion; Mellor and Cockburn, 1986). Rectal temperature has been found to be positively correlated with the amount of colostrum consumed (Devillers et al., 2011), as adequate colostrum consumption can increase body thermogenesis by approximately 30% (Mellor and Cockburn, 1986). Our data support this finding as COL20 had the highest average 24-h rectal temperature and COL10 had the lowest average rectal temperature.

Piglets that consume energy at levels higher than what is required for maintenance and thermogenesis will use resources to support body growth. In our study, there was a larger increase in weight gain over the first 24 h in COL20 and COL15 compared to COL10. Thus, the COL20 group received enough colostrum to satisfy maintenance energy needs and was able to divert a larger amount of energy into body growth than COL15 and COL10. The average increase in body mass of COL10 was only 8.8 g, whereas the average 24 h weight gain was 10-fold higher in COL15 gilts and more than 15-fold higher in COL20 gilts. The difference in 24-h growth rate carried over to the trajectory of growth, with COL15 and COL10 animals exhibiting lower growth response curves than COL20. Although growth response curves were different, ADG between PND 2 and PND 7 was not different between treatments, despite that, between days 1 and 2, COL15 and COL20 lost weight, whereas the COL10 group gained or maintained body weight. Loss of weight in the COL15 and COL20 groups was likely due to learning how to suckle and compete for access to teats, upon return to litters of origin, whereas COL10 group maintained their relatively small rate of gain despite learning to suckle and potential competition. Previous studies (Devillers et al., 2011; Declerck et al., 2016) have found the effects of early colostrum consumption to have lasting effects on piglet growth trajectory. In our study, COL20 remained the highest weight group through PND 7. This highlights the importance of adequate colostrum consumption within the first 24 h of postnatal life, as it sets the growth trajectory of the piglet.

A limitation of this model is that piglets were only followed until PND 7, as they were euthanized as part of the larger study. Despite this limitation, comparison of growth trajectories across differing 24 h postnatal intake levels demonstrated that long-term growth rate is established within the first 24 h (Devillers et al., 2011). Another limitation of this model is the need to obtain and preserve colostrum from multiple sows so that a pooled sample can be created. Freezing–thawing cycles alters the quality and potential bioactivity of multiple components in the colostrum, such as immunoglobulins (Argüello et al., 2003). To limit this potential and maintain colostrum quality, samples were exposed to only one freeze–thaw cycle prior to feeding gilts.

## CONCLUSION

Standardized doses of colostrum intake based on percentage of birth body weight over the first 24 h postnatal had distinct effects on 24-h weight gain, rectal temperature, and blood plasma immunocrit levels. Piglets consuming 20% of birth weight in colostrum were able to consume enough energy and nutrients to increase their body mass and temperature to satisfy their maintenance needs and use energy for growth. Piglets consuming 10% of birth weight in colostrum met basal maintenance needs for survival, but body temperature was compromised and 24 h growth was minimal. Importantly, 24-h colostrum doses of 10% and 20% birth body weight established significantly different growth trajectories over the first week postnatal. Trajectories of growth in the first week postnatal have been shown to be carried through to maturity and are related to long-term carcass composition, feed efficiency, and fertility. Therefore, using a homogenous colostrum sample that is administered based on birth body weight can be used as a research model to study the quantitative effects of varied amounts of 24-h postnatal colostrum intake on the long-term development of swine.
